# Books and Magazines

**Published:** 1909-02

**Authors:** 


### Books and Magazines.

---

SURGICAL MEMOIRS. By James G. Mumford, M. D., Instructor in Survery, Harvard Medical School; Visiting Surgeon to the Massachusetts General Hospital; Fellow of the American Surgical Association, etc., etc. Illustrated. $2.50 net. Moffat, Yard & Company, New York.

In this volume of collected essays, Dr. Mumford reproduces many of his papers and addresses of the last ten years, and adds some material hitherto unpublished. Mainly, the author deals with the History and Philosophy of Medicine.

The first essay is a narrative sketch of the History of Surgery, and embraces accounts of the great heroes of that art: Hippocrates, Galen, Vesalius, Paré, Haller, John Hunter and Lister. Then follows a paper, summing up ancient surgical accomplishments; succeeded by biographical essays on Cooper, Brodie, J. C. Warren, Bigelow. The remaining papers in the book are fugitive essays; accounts of special American achievements in medicine; a critical and historical essay on aneurism; addresses to nurses; and short papers on ethics and on medical education.

---

PRACTICAL POINTS IN ANESTHESIA. By Frederick-Emil Neef, B. S., B. L., M. L., M. D., New York. Price, Semi-De Luxe-Cloth, 60 cents, postpaid. Library. De Luxe Ooze Flexible leather, $1.50, postpaid. Surgery Publishing Co., 92 William Street, N. Y., U. S. A.

This very practical monograph presents the author's impressions on the correct use of chloroform, ether, etc., and is a simple and coherent working method, and is of particular value to those general practitioners who are so situated that the services of a trained anesthetist can not be secured.

This extremely practical and useful little book is condensed to about fifty pages, but every page is replete with valuable data. Printed upon heavy India Tint Special Cheltenham paper with Cheltenham type, with marginal headings in contrasting colored ink.

MODERN MEDICINE—ITS THEORY AND PRACTICE.—In Original Contributions by American and Foreign Authors. Edited by William Osler, M. D., Regius Professor of Medicine in Oxford University, England; formerly Professor of Medicine in Johns Hopkins University, Baltimore; in the University of Pennsylvania, Philadelphia, and in McGill University, Montreal. Assisted by Thomas McCrea, M. D., Associate Professor of Medicine and Clinical Therapeutics in Johns Hopkins University, Baltimore. In seven octavo volumes of about 900 pages each, illustrated. Volume V, Diseases of the Alimentary Tract. Just ready. Price per volume: cloth, $6.00 net; leather, $7.00 net; half morocco, $7.50 net. Lea & Febiger, Publishers, Philadelphia and New York, 1908.

This great work goes steadily forward to completion, the fifth of the seven volumes now being fresh from the press. It covers the great field of Diseases of the Digestive System and furnishes a thoroughgoing and authoritative exposition of a group of primary importance. The convenience of having the whole of the great divisions of disease in single volumes has evidently been borne in mind, and the same idea of logical classification and arrangement has been carried out even to paragraphing, so that any desired item of information can be quickly found. Nothing could be simpler or better than the uniform presentation of each disease in sections dealing with the cause, pathology, symptoms, diagnosis, course and prognosis, and treatment. The paramount importance of the latter is recognized in the fullness with which it is considered.

Modern Medicine differs from anything undertaken in the past in at least one very important particular, namely, its cosmopolitanism. The world is a unit in these days of quick communication, a fact that is vastly beneficial to its inhabitants. The leaders of medicine are scattered through all civilized countries, but engaged in the same quest of knowledge wherewith to combat disease. This knowledge would be confined within very small circles were it not for some means of diffusion, such as Modern Medicine, which carries it to all who read English—a large section of mankind. Professor Osler has distinguished himself both as a great physician and a great editor. He not only knows medicine and is interested in every part of it, but also knows the men who are doing the best work everywhere. Consequently, he has been able to select the best authority for each subject, and, moreover, to secure his co-operation. Such is the advantage of prestige and position.

It is scarcely necessary to point out the benefit which every physician can derive from possessing and consulting a work covering the entire domain of practical medicine and reflecting the world's latest and best knowledge. In these pages he can qualify as never before to meet his responsibilities.

